# American Medical Association

**Published:** 1851-03

**Authors:** 


### ARTICLE XIII.

### *American Medical Association.*

The Committee of Arrangements request all societies and other institutions authorized to send delegates, to forward a correct list of those selected to attend the next annual meeting, to the Secretary, Dr. H. W. DeSaussure, at Charleston, S. C., on or before the 1st day of April.

In consequence of the resignation of Dr. Stille, one of the Secretaries, from ill health, all communications intended for the next meeting of the Association must be addressed to the remaining Secretary, Dr. H. W. DeSaussure, Charleston, South Carolina.

The Fourth Annual Meeting of the American Medical Association will be held at Charleston, S. C., on the 1st Tuesday of May uext.

Editors of Medical Journals will please give the above notice an early insertion in their respective journals.—*Charleston Medical Journal.*

